# Medical treatment for urogenital tuberculosis (UGTB)

**DOI:** 10.3205/id000039

**Published:** 2018-08-09

**Authors:** Christian Wejse

**Affiliations:** 1 Department of Infectious Diseases/Center for Global Health, Dept of Public Health, Aarhus University, Denmark

## Abstract

Urogenital tuberculosis (UGTB) should in general be treated as pulmonary TB with a four-drug regimen of Isoniazid, Rifampicin, Ethambutol and Pyrazinamide for a total of 6 months, Ethambutol and Pyrazinamide only the first two months. Some patients may need longer treatment (cavitary disease, kidney abscess/malfunction, HIV co-infection). Treatment of multi-drug resistant tuberculosis (MDR-TB) requires use of long-term intravenous treatment with aminoglycosides and other drugs with considerable toxicity for 18–24 months. Complications such as urinary tract obstruction may occur and should be treated with corticosteroids or surgery.

## Medical treatment

Treatment of UGTB is in general similar to treatment for pulmonary TB. Treatment according to WHO guidelines with four drugs is used: Isoniazid (INH), Rifampicin (RIF), Ethambutol (ETH) and Pyrazinamide (PYR) as displayed in Table 1 (Tab. 1) and Table 2 (Tab. 2). Standard treatment duration is two months of intensive phase with all four drugs daily, followed by 4 or 7 months of continuation phase with only two drugs (INH+RIF), in total 6–9 months. Appropriate treatment with standard antituberculous agents for six months is usually successful in eradicating active UGTB, provided that it is drug-susceptible TB [1], and this is the general recommended duration of treatment [2], [3]. The 4-month continuation phase should therefore be used in the large majority of patients, the 7-month continuation phase should be reserved only for special groups of patients, such as patients with cavitary pulmonary TB with a positive sputum culture after the initial 2-month treatment. TB is often a multiorgan disease, and in case of involvement of CNS or bone/joint, treatment should be prolonged to 12 months. This may also be useful in cases with extensive soft-tissue involvement [4]. In particular patients with major kidney involvement and abscess or compromised renal function may need prolonged 

treatment for 9–12 months. In case of concomitant HIV infection, TB treatment may also be prolonged in particular if the patient is severely immunocompromised, and HIV testing is obligatory prior to any initiation of antituberculous treatment. Some forms of anti-retroviral treatment (ART) for HIV will require modifications in the TB treatment regimen such as replacement of Rifampicin with Rifabutin. In case of concomitant HIV infection, an infectious disease specialist should be consulted for initiation of ART, and combining ART with the necessary TB treatment is a task for a specialist because of several interactions, and this is not outlined here.

## Treatment of multi-drug resistant tuberculosis

Treatment of multi-drug resistant tuberculosis (MDR-TB) defined as resistance to both RIF and INH is a particular difficult issue, requiring use of toxic and perhaps very expensive drugs. MDR-TB constitutes globally 3.9% of new TB cases and 21% of previously treated cases [5], this equals to 580,000 new cases annually and an estimated 250,000 deaths from MDR-TB [5], unfortunately only 20% of those in need of MDR-TB treatment are enrolled on an MDR-TB treatment program. It is very important that all patients undergoing TB treatment have had samples sent for TB culture prior to treatment in order to be able to do resistance testing.

ln case of multi-drug resistance, the treatment should include the use of at least five drugs assumed to be effective, preferably documented through drug susceptibility tests. Treatment of MDR-TB is very complicated and has a duration of up to two years. In case of drug resistance, an infectious disease specialist with experience in MDR-TB treatment should be consulted, as it is generally kept on few hands because of the considerable toxicity of the drugs. Briefly the main aspects of MDR-TB treatment are outlined here, as MDR-TB constitutes up to 30% of new TB cases and 70% of re-treatment cases in some countries in Eastern Europe [6]. Treatment of MDR-TB may therefore be a very common issue faced in UGTB treatment in some areas. If possible, an expert consilium should be consulted [7]. Globally, only 50% of all MDR-TB cases are successfully treated [5]. If an MDR-TB case is not successfully treated, there is a risk of progression to XDR-TB (extensively drug resistant TB) which means additional resistance to aminoglycosides and fluorquinolones. These patients are even more difficult to treat, hence expert consultations for treatment is obligatory. XDR-TB has been reported in 117 countries and an estimated 9.5% of all patients with MDR-TB have XDR-TB [5].

The main concept in treatment of MDR-TB [8] is to use an injectable agent such as amikacin, kanamycin, or capreomycin (streptomycin is usually not used because of high rates of resistance), a fluoroquinolone (levofloxacin, moxifloxacin or gatifloxacin are recommended), and at least three other agents with probable activity (ethionamide or prothionamide, cycloserine, para-aminosalicylic acid). First line agents (PYR and ETH) with retained activity should also be used. Patients with MDR-TB require a regimen with at least five effective TB medicines during the intensive phase; PYR and four core second-line TB drugs (see Table 3 (Tab. 3)) – one each from Group A and Group B, and at least two from Group C [9], [10].

Ideally the injectable agent is administered daily for the first 6–8 months, forming an “intensive phase” of treatment, with the other drugs then continued, forming a “continuation phase” which should last an additional 12–16 months. There is a short course treatment recommended for selected cases, but since extra-pulmonary TB patients are never considered for this, it does not apply to UGTB patients [10]. Very often, adverse effects (nephro- and ototoxicity) will require ceasing treatment with the injectable agent, but it is often possible to manage side effects through dose reduction to 3 times weekly or swapping drugs for remaining alternatives.

In patients with MDR-TB, surgery may also be used as a means to reduce the amount of lung tissue with intractable pathology, to reduce bacterial load and thus improve prognosis [11]. WHO now recommends elective partial lung resection (lobectomy or wedge resection) alongside a recommended MDR-TB regimen [10].

## Treatment variations according to species

UGTB is usually caused by *Mycobacterium tuberculosis* or (in particular in West Africa) by* Mycobacterium africanum*. However, *Mycobacterium bovis* is also part of the *Mycobacterium tuberculosis *complex and may be seen in UGTB, in particular among elderly patients. Non-tuberculous mycobacteria are rare causes of urinary tract disease, and are not considered here. *M. bovis* is inherently resistant to PYR. This should therefore be avoided in such cases, and could be replaced with a flouorquinolone.

## Renal impairment

In case of renal insufficiency, drug dose adjustments are required for ETH and PYR as these drugs are cleared in the kidneys, INH and RIF can safely be used without dosing adjustments, even in end-stage renal disease. Dose-adjustments are made according to Glomerular Filtration Rate (GFR), as shown in Table 4 (Tab. 4). For these particular cases, monitoring plasma drug-concentrations is useful if available. PYR may induce hyperuricaemia and hyperuricuria (which may also be used to monitor treatment adherence) but could therefore be harmful in case of urinary tract damages. In such cases, a xanthine oxydase inhibitor (Allopurinol) could be added [12].

## Treatment response

The clinical response to antituberculous treatment is usually excellent because of high urinary concentrations of antituberculous drugs and good renal vascular supply. Sterilization of mycobacteria in urine usually occurs within two weeks after treatment start. Yet, there is often a considerable mortality, just as in TB disease in other organs. This is mostly because of late presentation or co-morbidity, and the overall TB mortality in 2015 was 14.5% [5]. In a UGTB cohort from Thailand the mortality was 26%, with age, comorbidity and no treatment being the major risk factors [13]. In other cohorts, mortality as low as 1% has been reported [14]. Relapse of TB is rare in culture-confirmed urinary tract disease treated with standard therapy [15], even among patients who require nephrectomy [16]. There are also some reports of high relapse rates, up to 20% in Turkey [17], [18], but historical cohorts with long follow-up >10 years indicate that cure rates of close to 100% are generally to be expected on current treatment regimens [19]. 

## Complications during treatment

During antituberculous therapy upper urinary tract obstruction (UTO) may occur [20]. Signs and symptoms of UTO should be monitored (flank pain, renal colic, hydronephrosis) in order to detect this debilitating condition, which may occur in the first few weeks of antituberculous therapy. It may be interpreted as a form of paradoxical reaction to treatment, which is often seen in TB, in particular but not only among HIV-infected in combination with initiating antiretroviral therapy [2], [21]. It is caused by inflammation, followed by fibrosis and obstruction of the collecting system [16]. Ureteral strictures may be caused by the disease process prior to treatment, but may also progress during treatment due to scarring and subsequent narrowing of the lumen [3]. Usually, this is occurring within the first two months of treatment [22]. Fibrosis of the bladder wall with reduced capacity is seen in 9% of patients [16]. In severe and neglected cases, bladder contractions may even develop [23].

## Treatment of complications

The treatment for obstruction and other manifestations of paradoxical reaction may primarily be surgical and contractions may need major surgical reconstruction [23]. Some reports indicate a very frequent need for surgery, up to 32% of male cases with UGTB in a large India cohort [4]. When urinary tract obstructions occur, anti-tuberculous treatment is not always sufficient, and reconstructive surgery may be indicated [3]. When UGTB has led to major kidney lesions such as caverns, surgical intervention is indicated in advanced cases [24]. All surgical interventions should be performed under the coverage of anti-TB therapy. Adding corticosteroid treatment may be considered in particular in minor strictures, e.g. prednisolon 50 mg daily with gradual rundown over weeks. Supplementary treatment with corticosteroids is not generally recommended in UGTB [2], but there is no reason to fear adding corticosteroids, once the patient is on effective treatment. This will not put the patient at increased risk of disseminated disease and corticosteroid as adjunctive therapy is increasingly used even in pulmonary TB [25].

## Note

This article is also to be published as a chapter of the Living Handbook „Urogenital Infections and Inflammations“ [26].

## Competing interests

The author declares that he has no competing interests.

## Figures and Tables

**Table 1:**
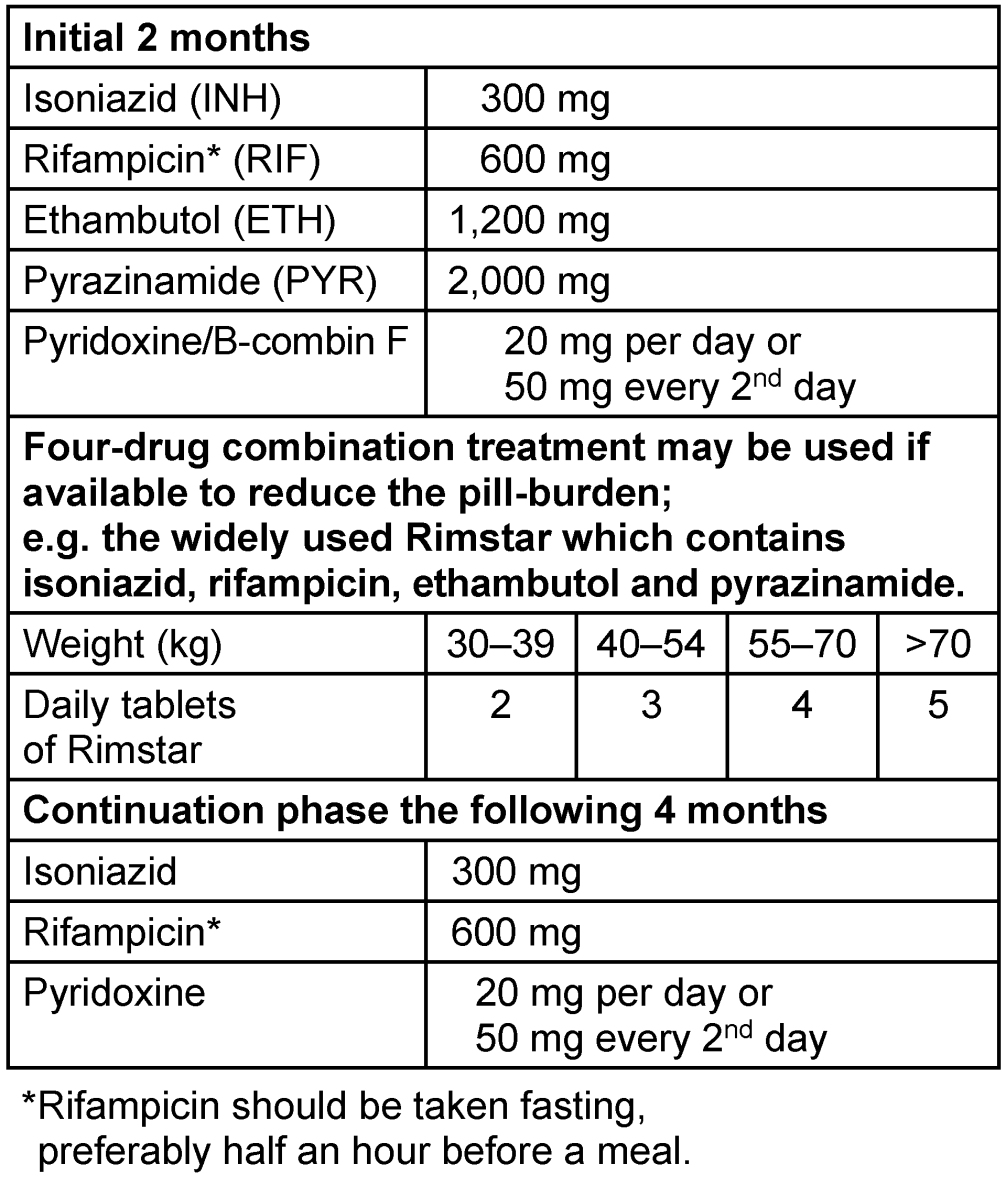
Treatment regimen for UGTB

**Table 2:**
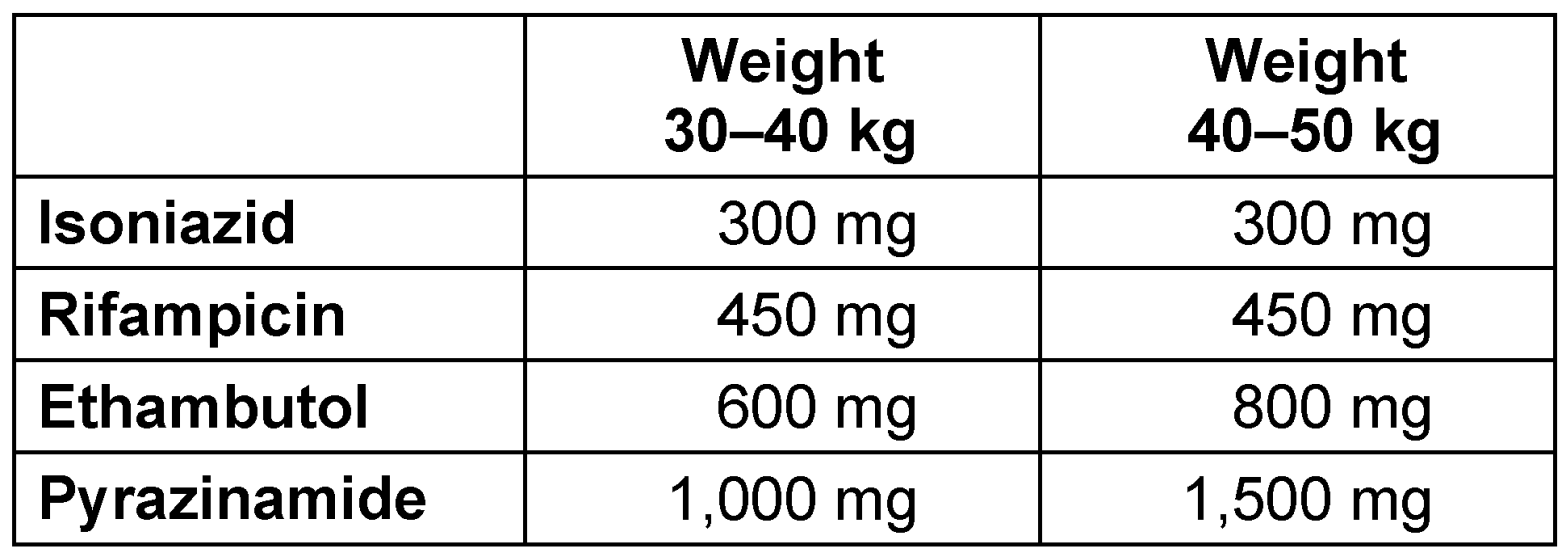
Dose-reduction according to weight (adults <50 kg)

**Table 3:**
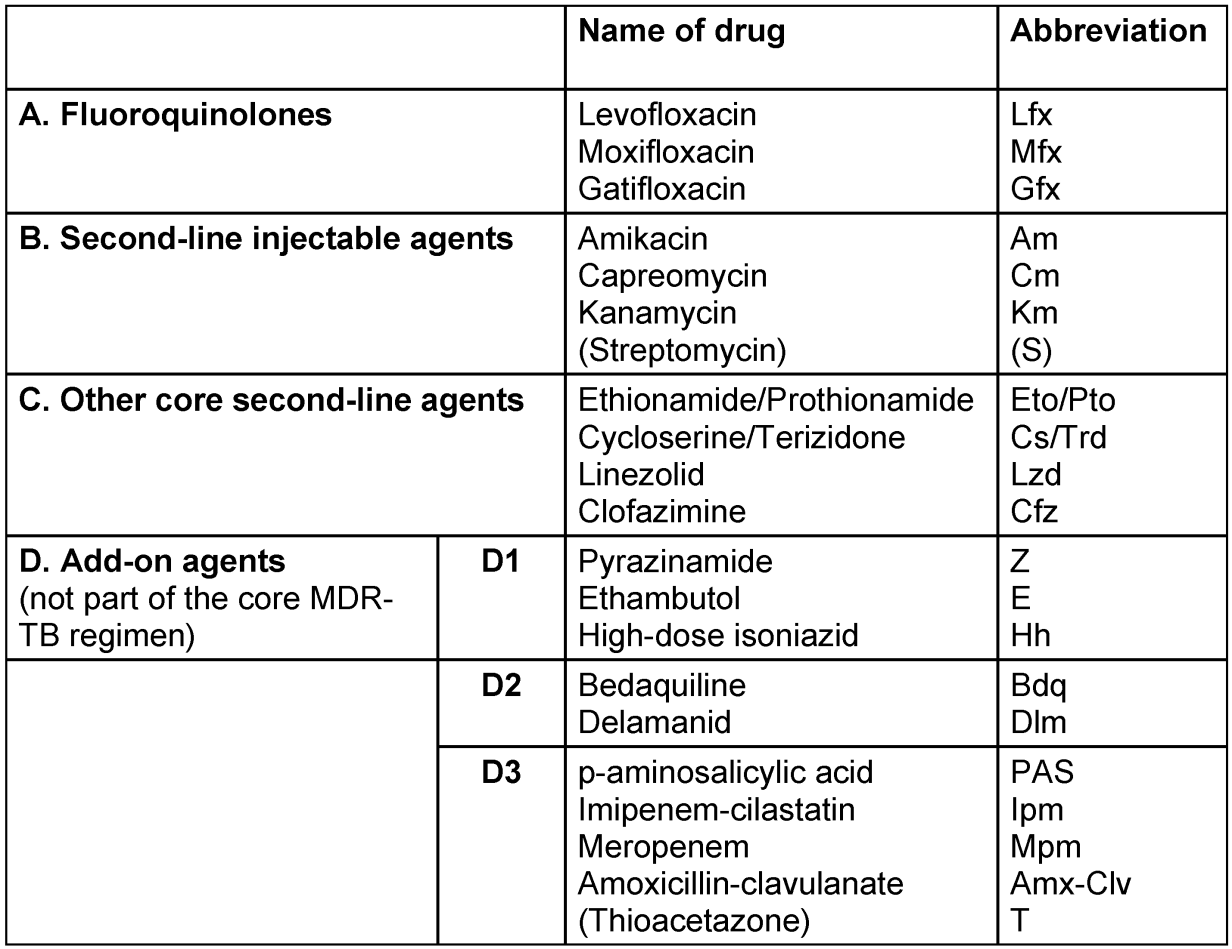
Drugs used for treatment of multi-drug resistant tuberculosis (MDR-TB)

**Table 4:**
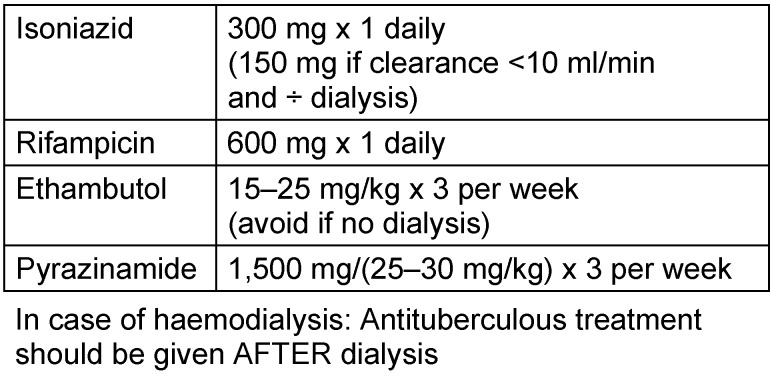
Reduced kidney function (creatinin clearance <30 ml/min); dose-reduction, adults
